# Complete Genome Sequences of a Cytopathic/Noncytopathic Pair of Border Disease Viruses

**DOI:** 10.1128/genomeA.00260-14

**Published:** 2014-04-10

**Authors:** John D. Neill, Julia F. Ridpath

**Affiliations:** Ruminant Diseases and Immunology Research Unit, National Animal Disease Center, U.S. Department of Agriculture, ARS, Ames, Iowa, USA

## Abstract

The complete genome sequences of a cytopathic/noncytopathic pair of border disease viruses (BDV) that were isolated from a sheep with mucosal disease-like lesions were determined. The cytopathic virus possessed an insert of 288 nucleotides that was derived from the Jiv cellular transcript and had 37 nucleotide differences from the noncytopathic virus.

## GENOME ANNOUNCEMENT

Border disease virus (BDV), a member of the *Pestivirus* genus of the *Flaviviridae*, is the causative agent of border disease (BD) in sheep and goats. BD is characterized by abortions, stillbirths, congenital deformities, and weak lambs. Like bovine viral diarrhea virus (BVDV), BDV can be classified as noncytopathic (ncp) or cytopathic (cp) based on its growth characteristics *in vitro*. For BVDV, the cp form arises from the ncp virus by insertions of cellular sequences or rearrangement and duplication of viral sequences (1–5). When infection of a pregnant animal occurs in the first trimester of pregnancy with an ncp virus, the fetus can become persistently infected (PI). Superinfection of the PI animal with a related cp virus results in a generally lethal disease, called mucosal disease. A similar scenario for BDV is assumed.

The BDV strains Coos Bay 5 ncp (CB5nc) and cp (CB5c) were isolated and biologically cloned in the late 1980s from a sheep with mucosal disease-like lesions. Viral RNA was extracted following treatment of the virus stocks with a nuclease cocktail (6). Both viruses were sequenced using a random-primed, single-primer method for synthesis of bar-coded, double-stranded cDNA (7). Briefly, a single-tube reverse transcriptase, second-strand cDNA synthesis reaction was done using primers with 20 bases of known sequence and 8 random nucleotides on the 3′ ends. The cDNA was amplified using primers matching the 20mer sequence, and the amplicons were prepared for sequencing using the Ion Torrent sequencing platform (Life Technologies, Inc., Grand Island, NY). The sequencing run was demultiplexed using the 20 base sequences (7). The genomes were assembled with SeqMan NGen software (DNAstar, Inc., Madison, WI) using BDV strain X818 (GenBank accession number AF037405) as the template. The 5′ and 3′ ends of the genomic RNAs were confirmed by sequencing of PCR products using primer sets designed to amplify the 600 5′ bases and the 1,000 3′ terminal bases.

The libraries yielded 13,755 (8,762 virus) and 41,679 (27,486 virus) sequences for CB5nc and CB5c, respectively. The genomes of CB5nc and CB5c were 12,300 and 12,588 bases. The single open reading frame (ORF) of CB5nc was 11,694 bases (3,898 amino acids) and the ORF of CB5c was 11,982 bases (3,993 amino acids). CB5c had a 288-base insertion derived from the Jiv cellular transcript (8, 9) in the NS2/NS3 coding region. Similar inserts were observed in BVDV type 2 cp genomes (1, 10). Excluding the insert, the two viruses had 37 nucleotide differences and 11 amino acid changes. Four of these amino acid differences (36%) were found in the immunodominant E2 structural protein. Amino acid changes introduced into the E2 protein of BVDV type 1 in the establishment of a persistent infection can result in antigenic changes in the protein (11). Additionally, both viruses had multiple, small deletions with a total of 33 bases deleted from the 3′ untranslated region (UTR) compared to X818 and BD31 (GenBank accession number U70263). This is the first report of a border disease virus noncyptopathic/cytopathic pair that was isolated from a sheep with mucosal disease-like lesions.

### Nucleotide sequence accession numbers.

The genomic sequences of the Coos Bay noncytopathic and cytopathic viruses have been deposited in GenBank with the accessions numbers KJ463422 and KJ463423, respectively.
